# l-Phenylalanine Restores Vascular Function in Spontaneously Hypertensive Rats Through Activation of the GCH1-GFRP Complex

**DOI:** 10.1016/j.jacbts.2018.01.015

**Published:** 2018-05-30

**Authors:** Lamia Heikal, Anna Starr, Dania Hussein, Jesus Prieto-Lloret, Phil Aaronson, Lea Ann Dailey, Manasi Nandi

**Affiliations:** a Institute of Pharmaceutical Sciences, Faculty of Life Sciences and Medicine, King’s College London, London, United Kingdom; b Division of Asthma, Faculty of Life Sciences and Medicine, King’s College London, London, United Kingdom; c Cardiovascular Division, Faculty of Life Sciences and Medicine, King’s College London, London, United Kingdom

**Keywords:** cardiovascular disease, endothelium, l-phenylalanine, nitric oxide, tetrahydrobiopterin, vascular activity, ACh, acetylcholine, ANOVA, analysis of variance, BH_2_, dihydrobiopterin, BH_4_, tetrahydrobiopterin, EC_50_, effective concentration for 50% maximal response, EDHF, endothelium derived hyperpolarizing factor, eNOS, endothelial nitric oxide synthase, GCH1, GTP cyclohydrolase-1, GFRP, GCH1 feedback regulatory protein, L-phe, l-phenylalanine, L-tyr, l-tyrosine, NO, nitric oxide, ROS, reactive oxygen species, SHR, spontaneously hypertensive rat(s), WKY, Wistar Kyoto rat(s)

## Abstract

Reduced nitric oxide (NO) bioavailability correlates with impaired cardiovascular function. NO is extremely labile and has been challenging to develop as a therapeutic agent. However, NO bioavailability could be enhanced by pharmacologically targeting endogenous NO regulatory pathways. Tetrahydrobiopterin, an essential cofactor for NO production, is synthesized by GTP cyclohydrolase-1 (GCH1), which complexes with GCH1 feedback regulatory protein (GFRP). The dietary amino acid l-phenylalanine activates this complex, elevating vascular BH_4_. Here, the authors demonstrate that l-phenylalanine administration restores vascular function in a rodent model of hypertension, suggesting the GCH1-GFRP complex represents a rational therapeutic target for diseases underpinned by endothelial dysfunction.

## Highlights

• Tetrahydrobiopterin is an essential cofactor for NO production.
• Limitation of endogenous tetrahydrobiopterin reduces NO bioavailability, enhances oxidative stress, and impairs vascular function.
• Orally supplemented tetrahydrobiopterin has therapeutic challenges because it is rapidly oxidized in vivo.
• Here, the authors demonstrate that l-phenylalanine, when administered orally, raises vascular tetrahydrobiopterin, restores NO, reduces superoxide, and enhances vascular function in spontaneously hypertensive rats.
• This effect is achieved by activation of a protein complex (GCH1-GFRP) involved in the biosynthesis of tetrahydrobiopterin.
• Activation of this protein complex by l-phenylalanine or its analogues represents a novel therapeutic target for vascular disorders underpinned by reduced NO bioavailability.

## Visual Abstract

**Figure:**
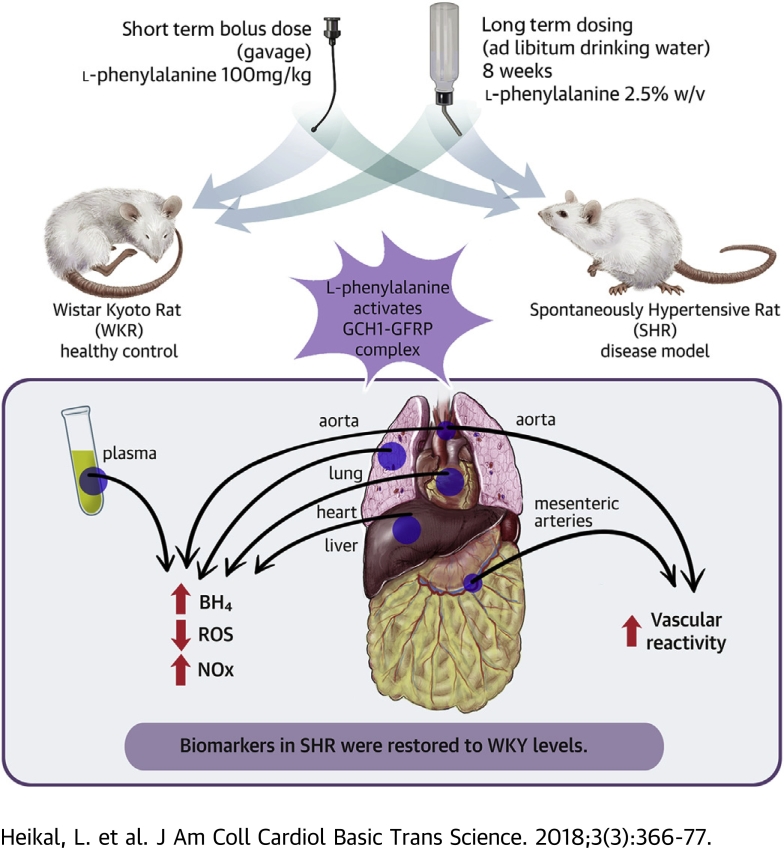

Cardiovascular diseases pose a considerable societal and economic burden on health care systems (1). Such diseases are usually associated with functional and structural changes within the vascular network as well as concomitant increases in oxidative stress (2). Endothelial dysfunction is characterized by impaired vasodilation, mainly due to loss of nitric oxide (NO) signaling 3, 4. NO biosynthesis in the vasculature is primarily catalyzed by endothelial nitric oxide synthase (eNOS) (5), and tetrahydrobiopterin (BH_4_) is an essential cofactor for all 3 isoforms of NOS 6, 7. When BH_4_ bioavailability is limited, NOS may become “uncoupled,” producing superoxide at the expense of NO, thereby potentiating oxidative stress (8). Thus, raising endothelial BH_4_ levels has been suggested as a strategy to maintain healthy NO production and bioavailability at the level of the endothelium 9, 10. To support this, intra-arterial administration of BH_4_ improves endothelial dysfunction in patients with hypertension (11), coronary artery disease (12), and hypercholesterolemia (13). However, due to its unstable nature, orally administered BH_4_ (or its analogue sapropterin) has limited efficacy in improving vascular hemodynamics 14, 15, 16. Therefore, other strategies to raise endogenous BH_4_ bioavailability at the level of the endothelium are desirable.

BH_4_ is synthesized from GTP in a reaction where the committing step is mediated by GTP cyclohydrolase-1 (GCH1) (17). Modulation of GCH1 expression has been shown to regulate BH_4_, NO, and cardiovascular function 18, 19, 20. GCH1 is subject to feed-forward regulation by l-phenylalanine (L-phe), via an allosteric protein interaction with GCH1 feedback regulatory protein (GFRP) 21, 22. This GCH1-GFRP complex is operative in humans because oral challenge with L-phe leads to a 3-fold rise in plasma biopterin levels (a correlate of BH_4_)—an effect that is attenuated in patients with a loss-of-function GCH1 mutation (23). Targeting endogenous BH_4_ biosynthesis, by activating the GCH1-GFRP axis pharmacologically, thus represents a method to enhance vascular BH_4_ levels at the level of the endothelium, circumventing the poor bioavailability following oral BH_4_ administration 14, 24.

To support this hypothesis, it is known the GCH1-GFRP axis regulates BH_4_ and NO in endothelial cells (25). Overexpression of GFRP reduces basal BH_4_ levels (26) and attenuates the rise in BH_4_ and NO that occurs in response to a proinflammatory stimulus (27). Additionally, the primary source of BH_4_ appears to be derived from GCH1 localized within the vascular endothelium, and GFRP is coexpressed within these cells 28, 29. Finally, oral L-phe elicits a rise in vascular BH_4_—an effect that is absent in mice lacking endothelial GCH1 (24).

The GCH1-GFRP axis thus ensures that BH_4_ levels are kept within a tight physiological range. However, the crucial mechanistic link between GCH1-GFRP activation by L-phe, the concomitant rise in BH_4_ and its potential impact on NO and vascular function is lacking. The aim of this study was to determine whether L-phe raises vascular BH_4_ levels by activating the GCH1-GFRP complex in vivo and improves endothelial function in an animal model of essential hypertension.

## Methods

Further details of all assays can be found in the Supplemental Methods.

### Effect of L-phe on recombinantly expressed GCH-1 activity

A kinetic microplate assay was used to determine the effects of L-phe (1 mmol·l^−1^) on the activity of recombinantly expressed human GCH1 (0.1 μmol·l^−1^) either alone or when coincubated with recombinantly expressed human GFRP (1 μmol·l^−1^) (24). This assay measures the accumulation of the intermediate reaction product, dihydroneopterin triphosphate (H_2_NTP), by monitoring an increase in A_340_ over time (30) (Supplemental Methods).

### Animals

Spontaneously hypertensive rats (SHR) and Wistar Kyoto rats (WKY) were used throughout.

All animal experiments were performed under U.K. Home Office approval according to the Animals Scientific Procedures Act, 1986 and subsequent revisions and conformed to the Guide for the Care and Use of Laboratory Animals published by the National Institutes of Health (NIH Publication No. 85-23, revised 1996). Studies were designed and conducted in accord with the ARRIVE (Animals in Research: Reporting In Vivo Experiments) guidelines (31). The scientific rational for choice of animal, age group, and details of experimental design are fully described in the Supplemental Methods (31).

### L-phe oral challenge: Bolus dosing and long-term treatment

A short-term bolus dose of L-phe (100 mg/kg) or saline control was orally administered via gavage to 13-week-old WKY or SHR. Venous blood was collected, under brief inhaled isoflurane anesthesia, from the tail of each animal 0.5, 1, and 4 h after bolus dosing.

For assessment of the long-term effects of L-phe supplementation, 4-week-old SHR in their pre-hypertensive stage were given free access to drinking water supplemented with 2.5% w/v L-phe or saline until they reached 12 weeks of age, after which plasma and tissue samples were acquired (see the Supplemental Methods for L-phe dose calculation; Supplemental Figure 1 for justification of the time course of the long-term L-phe dosing study).

### BH_4_, dihydrobiopterin (BH_2_), and biopterin measurement

BH_4_, BH_2_, and biopterin were measured from snap-frozen whole tissue and plasma using fluorescence and electrochemical detection 32, 33, 34 (see the Supplemental Methods for full details).

### Nitrite measurement

Quantification of total NOx was performed as previously described using the modified Greiss assay (35) and fluorometric detection (36) (see the Supplemental Methods for full details).

### Quantification of superoxide levels

Superoxide levels were quantified using a lucigenin chemiluminescence-based assay as previously described (37) (see the Supplemental Methods for full details).

### Aromatic amino acid and catecholamine measurement

Phenylalanine, tyrosine, dopamine, and adrenaline/noradrenaline were measured by ultraviolet spectrophotometric detection in plasma and tissues (38) (see the Supplemental Methods for full details).

### Vascular reactivity of aortic rings

Experiments were carried out in fresh rat aortic rings ∼2 mm in length. Vessels were carefully dissected, and the endothelium was denuded using a steel wire in a proportion of rings. All intact and denuded aortic rings were suspended in an organ bath containing Krebs buffer, 5 μmol·l^−1^ indomethacin (a cyclooxygenase inhibitor) and gassed with 95% O_2_ and 5% CO_2_ at 37°C, as previously described (39). The presence of a functional endothelial cell layer was confirmed if a clear vasorelaxant response was observed to 1 μmol·l^−1^ acetylcholine (ACh) in tissues pre-contracted with 0.1 μmol·l^−1^ phenylephrine. Endothelium intact vessels that did not display >70% relaxation to the highest ACh dose were excluded from the study.

### Vascular reactivity of mesenteric arteries

Small resistance arteries (approximately 300 μm, length 3 to 4 mm) were isolated from SHR and WKY mesenteries, dissected free of surrounding fat and connective tissue, and mounted as isometric preparations on a Mulvany–Halpern wire myograph (Danish Myo Technology, Aarhus, Denmark) containing Krebs buffer (as in the preceding text). As before, the endothelial layer was intentionally removed in a proportion of the mesenteric rings. Vessels were stretched to a circumference 90% of that obtained when subjected to a transmural pressure of 13.4 kPa (40) before a routine “run-up” procedure consisting of 4 alternate contractions to high K^+^ solution (as in the preceding text). Endothelial viability was again assessed by the addition of 1 μmol·l^−1^ ACh to pre-contracted tissues.

### Effects of short-term exogenous L-phe on the vascular reactivity of aortic and mesenteric arteries from naive 13-week-old SHR and WKY

Following the initial vessel setup and endothelial integrity assessment (described in the preceding text), all blood vessels were contracted with effective concentration of phenylephrine for 80% maximal contraction (EC_80_) followed by a concentration response curve to ACh (0.01 to 1 μmol·l^−1^). Tissues were subsequently washed out and incubated with 0.5 mmol·l^−1^ L-phe or saline control, for 30 min. Post-incubation, vessels were recontracted with phenylephrine (EC_80_), and a second ACh concentration response curve was constructed. The EC_50_ values for ACh were recorded and compared in all vessels, pre- and post–L-phe incubation. Six aortic and 6 mesenteric rings were acquired from each animal. Three aortic/mesenteric rings were treated with L-phe, and the remaining 3 were controls (saline treated) in each experiment. Therefore, a total of 6 animals were used in 6 independent experiments, conducted in triplicate.

### Effects of long-term oral L-phe supplementation on the vascular reactivity of aortic and mesenteric arteries from 13-week-old SHR

To assess the effects of long-term L-phe on vascular reactivity, aortic rings, and mesenteric arteries were isolated from SHR treated long term with oral L-phe (8 weeks via drinking water) or saline control, as described earlier. Aortic and mesenteric rings were isolated and their integrity assessed as described in the preceding text. Vessels were pre-contracted with phenylephrine (EC_80_), and concentration response curves to ACh were constructed. The EC_50_ for ACh was recorded and compared between L-phe–treated SHR and compared with vehicle-treated SHR. Six aortic and 6 mesenteric rings were acquired from each animal, and a total of 6 animals/group were used in 6 independent experiments in triplicate.

#### Data acquisition

The changes in tension of all tissues/rings were measured using a force transducer, and responses were recorded and analyzed using LabChart software version 4.2 (ADInstruments Ltd., Oxford, United Kingdom) by a blinded investigator.

### Statistical analysis

All data were analyzed using GraphPad Prism software version 5 (GraphPad Software, La Jolla, California). Normal distribution of data was assessed followed by a Student’s *t* test or repeated-measures 1-way analysis of variance (ANOVA) followed by Bonferroni’s multiple comparisons post-test. Two-way ANOVA with Bonferroni post hoc test was used in the vascular activity experiments where the ANOVA compared the control (saline-treated) versus L-phe–treated curve for each experiment. EC_50_ values were compared using the Student’s *t* test. The following annotation system was used: *p < 0.05; **p < 0.01; and ***p < 0.001. Exact p values are listed where feasible in the figure, legend, or in the Supplemental Table 2.

## Results

### Effects of L-phe on recombinant GCH1 activity (in vitro) and systemic BH_4_ and nitrite (in vivo)

**Figure 1:**
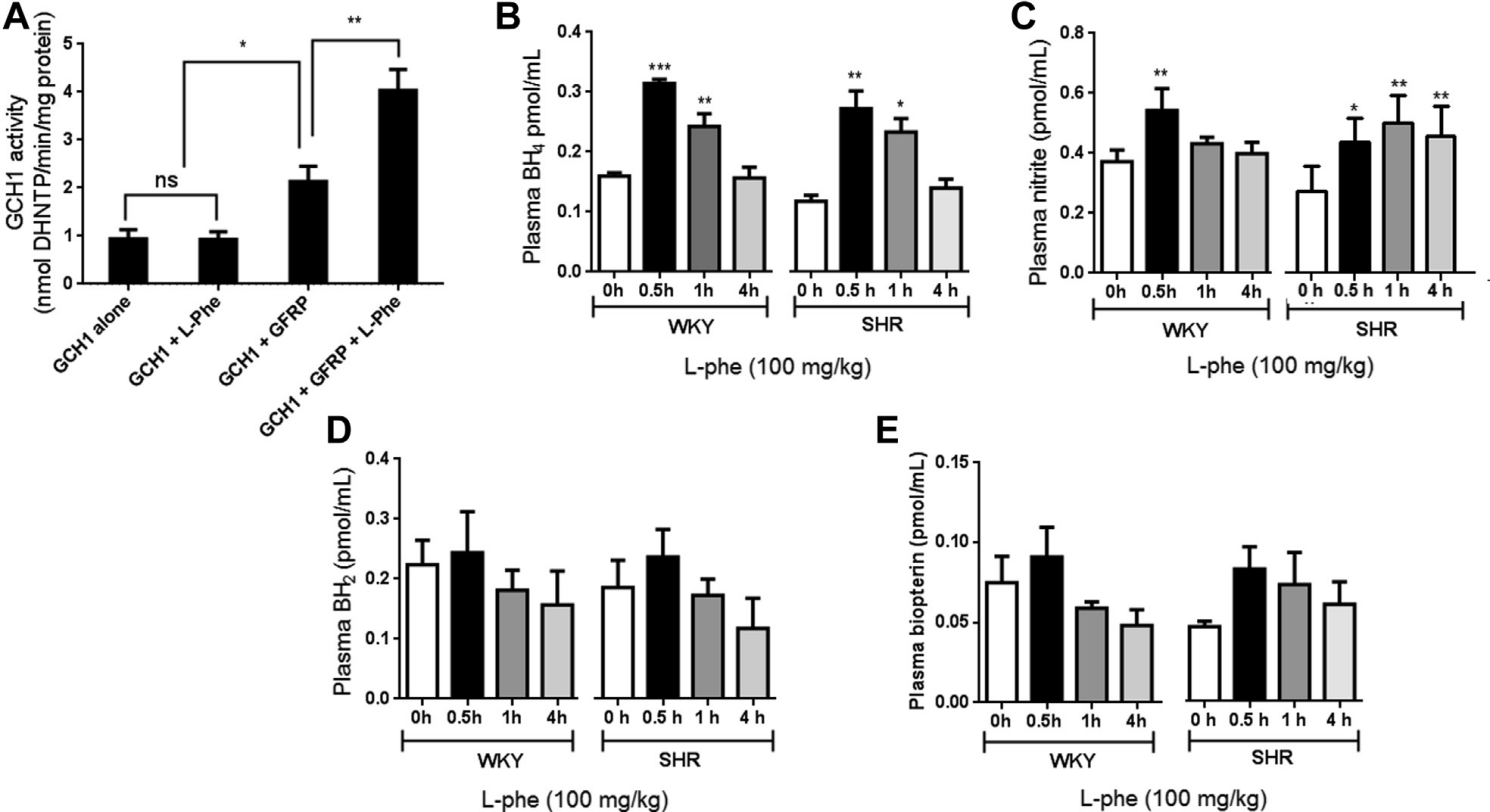
Effects of Short-Term L-Phe On Recombinant GCH1 Activity (In Vitro) and Systemic BH_4_ and Nitrite (In Vivo) **(A)** GCH1 activity, determined by the production of the intermediate product reaction; dihydroneopterin triphosphate (DHNTP) at 340 nm of purified GCH1 (0.1 μmol·l^−1^) alone or in the presence of purified GFRP (1 μmol·l^−1^) and/or L-phe (1 mmol·l^−1^). The datasets shown represent the mean ± SEM of n = 4 (*p < 0.05). Quantitative measurement of temporal changes in plasma **(B)** BH_4_, and **(C)** nitrite, **(D)** BH_2_, and **(E)** biopterin following 100 mg·kg^−1^ L-phe challenge in WKY and SHR (given via oral gavage). The datasets shown represent the mean ± SEM of n = 6 animals per group per time point compared with baseline control (0 h) (*p < 0.05, ** p < 0.01, ***p < 0.001). (Exact p values are tabulated in Supplemental Table 2). BH_2_ = dihydrobiopterin; BH_4_ = tetrahydrobiopterin; eNOS = endothelial nitric oxide synthase; GCH1 = GTP cyclohydrolase 1; L-Arg = l-arginine; L-phe = l-phenylalanine; SHR = spontaneously hypertensive rat(s); WKY = Wistar Kyoto rat(s).

As previously described 24, 30, the combination of purified recombinant GFRP with GCH1 protein had a higher basal activity than GCH1 alone in vitro. The addition of L-phe (2 mmol·l^−1^) had no effect on purified GCH1 activity alone but caused a significant rise in GCH1 activity when coincubated with GFRP, confirming that L-phe is an allosteric regulator of the GCH1-GFRP complex only (Figure 1A).

In vivo, oral L-phe (100 mg·kg^−1^) bolus to WKY and SHR significantly increased plasma BH_4_ levels within 30 min, and levels returned back to baseline within 4 h (Figure 1B). Correspondingly, a significant rise in nitrite levels was also detected within 30 min, but whereas this returned to baseline in WKY, it remained elevated in SHR for at least 4 h (Figure 1C). Interestingly, there were no statistically significant differences in BH_2_ and biopterin in all groups although trend increases were observed (Figures 1D and 1E).

### Effects of bolus and long-term L-phe on tissue BH_4_ and nitrite in vivo

**Figure 2:**
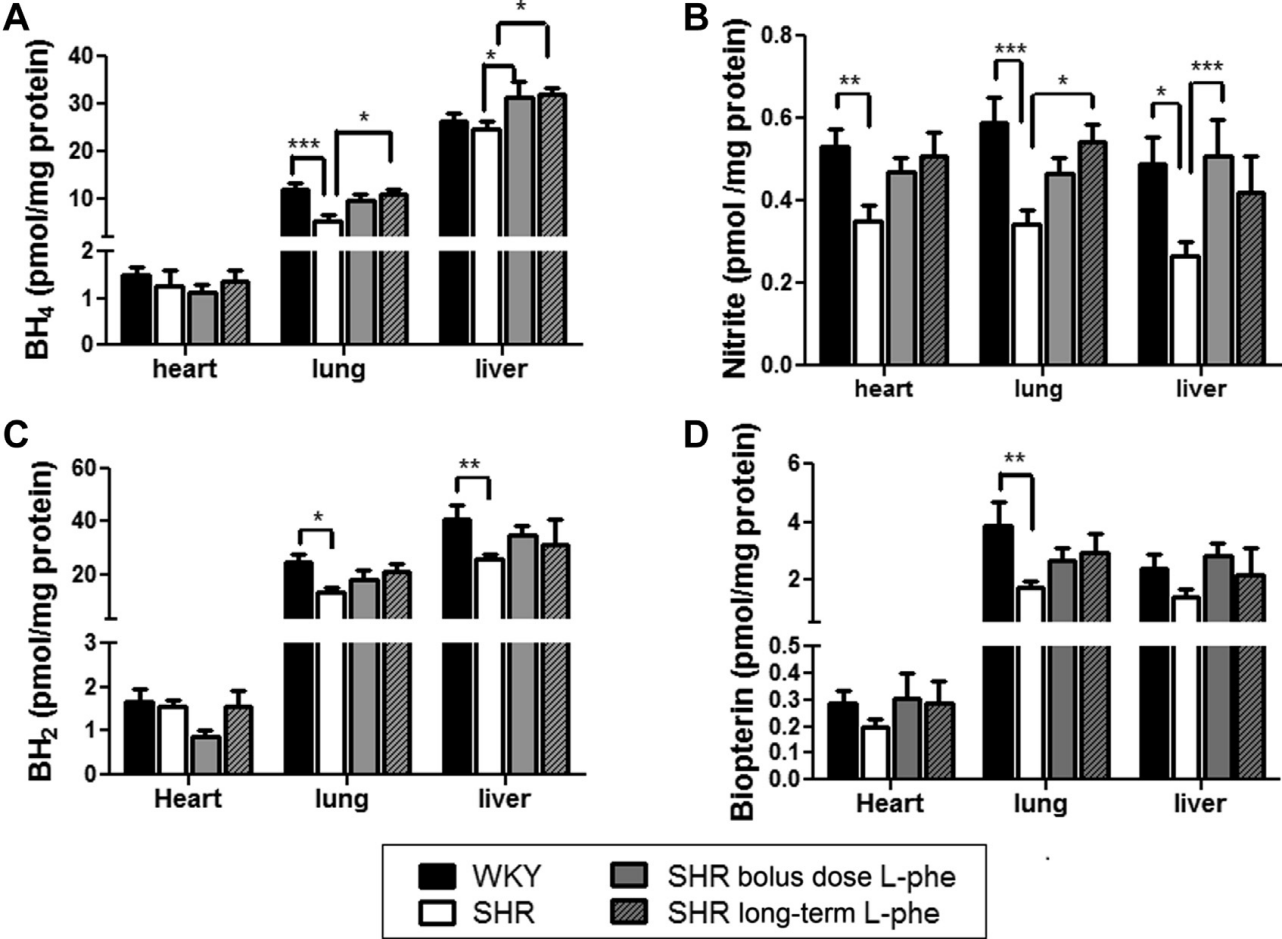
Effects of Short-Term and Long-Term L-Phe on Tissue BH_4_ and Nitrite In Vivo Short-term (100 mg·kg^−1^ L-phe, oral gavage, 4 h, n = 6) and long-term administration (2.5% L-phe in drinking water for 60 days, n = 6) in SHR on **(A)** BH_4_, **(B)** nitrite, **(C)** BH_2_, and **(D)** biopterin in heart, lung, and liver tissues. The datasets shown represent the mean ± SEM where *p < 0.05, **p < 0.01, and ***p < 0.001. (Exact p values are in the Supplemental Data.) Abbreviations as in Figure 1.

Baseline BH_4_, BH_2_, and biopterin levels were measured in plasma, heart, lung, and liver tissues from 13-week-old SHR and WKY. BH_4_, BH_2_, and/or biopterin were significantly reduced in lungs and plasma of SHR compared with WKY (Figures 2A, 2C, and 2D, Supplemental Figure 2). L-phe supplementation increased BH_4_ levels in lung and liver tissues in SHR in the short term (oral gavage, 4-h time point) and long term (drinking water, 8 weeks) (Figure 2A).

Nitrite levels were significantly lower in the heart, lung, and liver, but not the plasma, of SHR compared with WKY (Figure 2B, Supplemental Figure 2). Both bolus and long-term supplementation of L-phe normalized nitrite levels in SHR to WKY control values (Figure 2B). There were no statistically significant differences in BH_2_ or biopterin in all groups following bolus dose and long-term L-phe treatment, although trend increases were observed (Figures 2C and 2D, Supplemental Figure 2).

### Effects of L-phe on vascular BH_4_ and ROS

**Figure 3:**
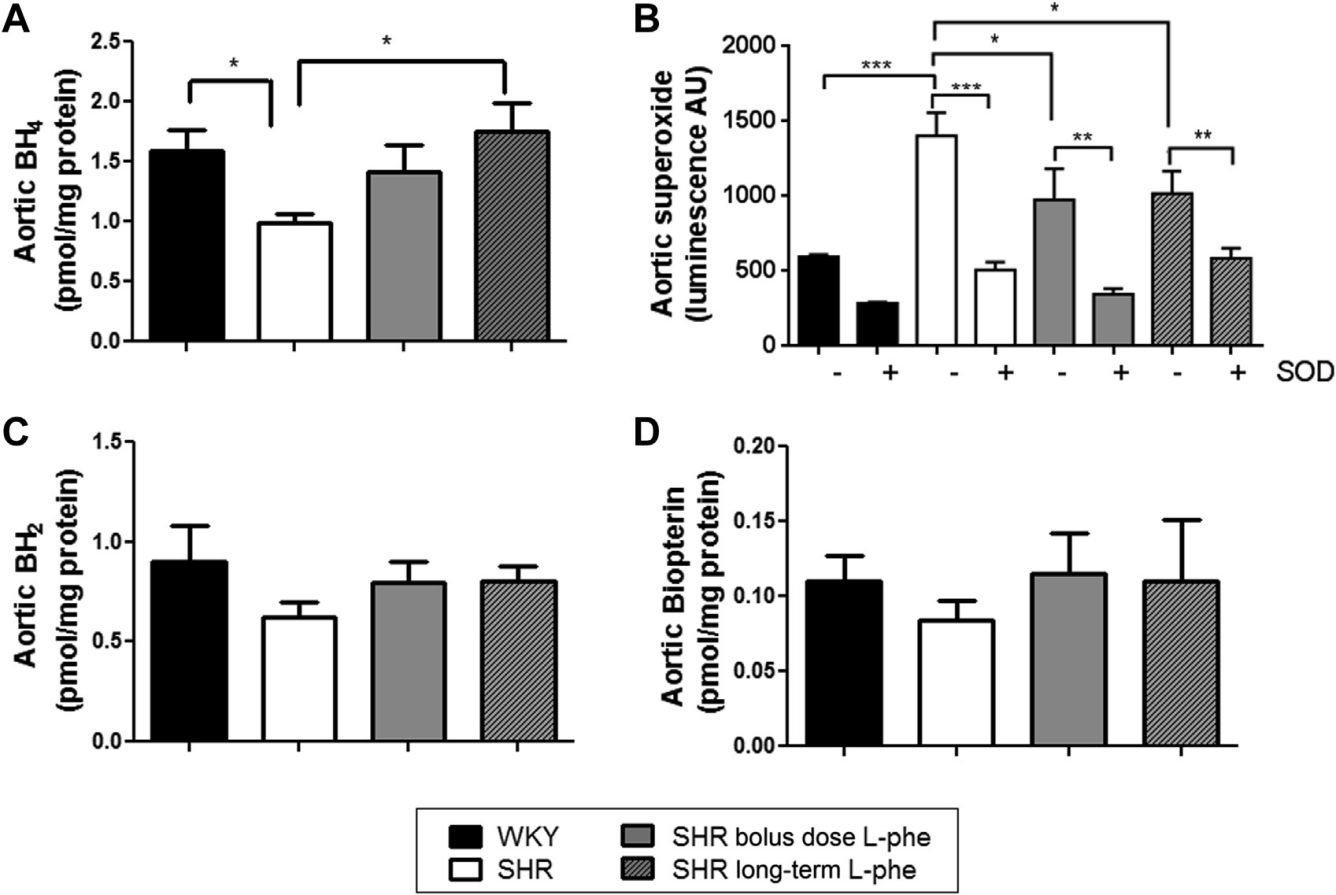
Effects of L-Phe on Aortic BH_4_ and ROS Effects of L-phe on aortic levels of **(A)** BH_4_, **(B)** superoxide, **(C)** BH_2_, and **(D)** biopterin after short-term (100 mg·kg^−1^ L-phe, oral gavage, 4 h) and long-term administration (2.5% L-phe in drinking water for 60 days, n = 6) in SHR. The datasets shown represent the mean ± SEM where *p < 0.05, **p < 0.01, and ***p < 0.001. Exact p values are in the Supplemental Data. ROS = reactive oxygen species; SOD = superoxide dismutase; other abbreviations as in Figure 1.

Further detailed studies in aortic tissue revealed a significantly lower BH_4_ level in SHR compared with WKY, which was restored to WKY values following short-term bolus and long-term L-phe treatment (Figure 3A). As anticipated, superoxide levels were higher basally in SHR compared with WKY (Figure 3B). Bolus dose or long-term administration of L-phe significantly reduced superoxide levels in SHR (Figure 3B). Superoxide dismutase, the positive control, reduced superoxide in all study groups (Figure 3B). Again, we observed no significant changes in BH_2_ or biopterin in aortic tissue ± L-phe administration (Figures 3C and 3D). Unfortunately, in aortic tissues, nitrite levels fell below the limit of detection and were therefore not quantifiable.

### Effects of short-term L-phe incubation on aortic and mesenteric vascular reactivity

**Figure 4:**
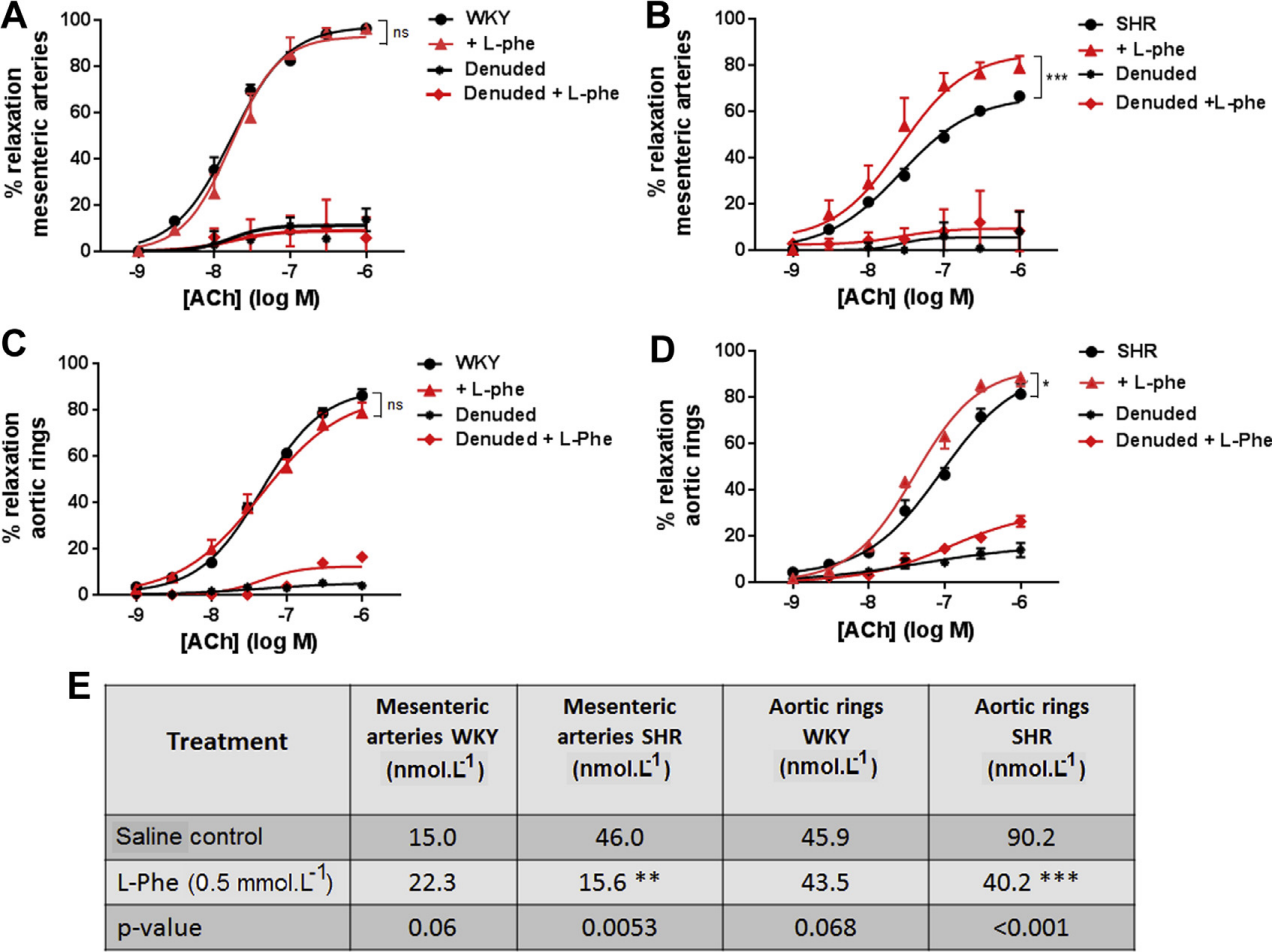
Effects of Short-Term L-Phe on Aortic and Mesenteric Vascular Reactivity Concentration response curves for acetylcholine in **(A)** WKY mesenteric rings, **(B)** SHR mesenteric rings, **(C)** WKY aortic rings, and **(D)** SHR aortic rings, with intact or denuded endothelium following short-term incubation with 0.5 mmol·l^−1^ L-phe. Data represent mean ± SEM n = 6 animals (in triplicate/animal) for mesenteric arteries and aortic rings (*p < 0.05 and *** p < 0.001 for the whole curve). Table **(E)** shows EC_50_ values (nmol·l^−1^) comparing saline control with L-phe treatment for each study. Data represent mean EC_50_ values ± SEM, n = 6 independent experiments (**p < 0.01 and ***p < 0.001). Ach = acetylcholine; EC_50_ = effective concentration for 50% maximal response; other abbreviations as in Figure 1.

Initial assessment of vascular responses showed that both the aortic and mesenteric vessels from SHR and WKY had similar contractile responses to phenylephrine, whereas endothelial dependent vasorelaxation to ACh was diminished in SHR compared with WKY (Figures 4A and 4C vs. 4B and 4D). Endothelial denudation conferred an 80% to 95% reduction of Ach-induced vasorelaxation, and there were no additional effects of L-phe, suggesting that it does not directly relax the smooth muscle (Figure 4). Following incubation with 0.5 mmol·l^−1^ L-phe, in the organ bath, contractile responses to phenylephrine were unaffected in both SHR and WKY vessels. Similarly, L-phe incubation had no effect on Ach-induced vasorelaxation in WKY vessels (Figures 4A and 4C). However, endothelial dependent relaxation to ACh was significantly improved by L-phe in aorta and mesenteric vessels from SHR, as reflected by the leftward shift of the dose-response curves (Figures 4B and 4D) and corresponding changes in the EC_50_ values (Figure 4C), an effect that was more pronounced in the mesenteric arteries compared with the aorta.

### Effects of long-term L-phe treatment on aortic and mesenteric vascular reactivity

**Figure 5:**
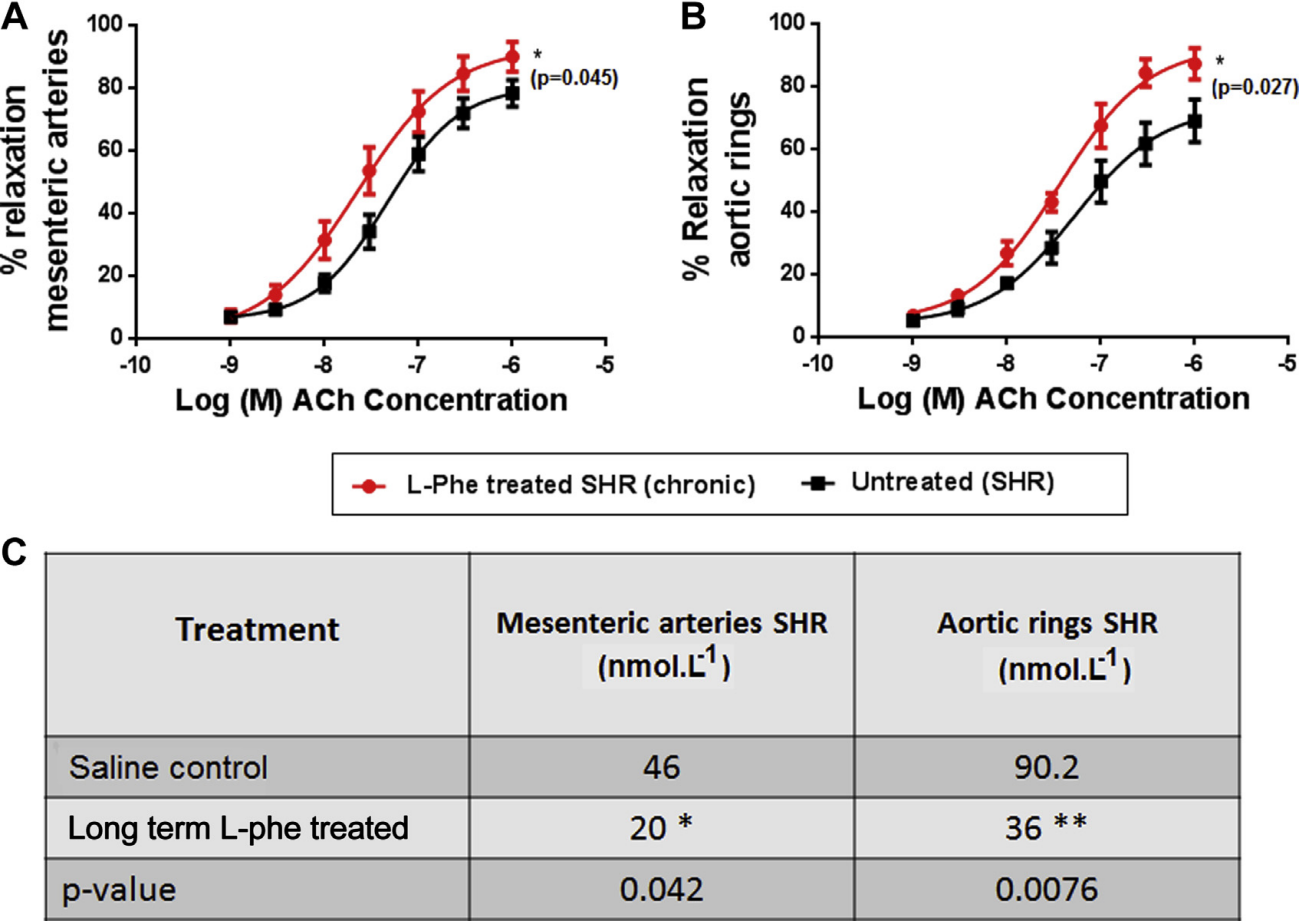
Effects of Long-Term L-Phe Treatment on Aortic and Mesenteric Vascular Reactivity Concentration response curves for acetylcholine in **(A)** mesenteric arteries and **(B)** aortic rings isolated from L-phe treated long-term treated SHR and saline controls. The table **(C)** shows corresponding EC_50_ values (*p < 0.05). Data represent mean ± SD, n = 6 independent experiments (*p < 0.05 for the whole curve). Abbreviations as in Figures 1 and 4.

Consistent with the effects of short-term L-phe incubation, endothelial-dependent relaxation to ACh in all vessels from SHR treated long-term with L-phe (8 weeks in drinking water) was significantly improved (p < 0.01) compared with saline control, as reflected by the leftward shift of the dose-response curves and change in the EC_50_ values (Figure 5C).

### Effects of bolus dose and long-term L-phe on systemic and tissue phenylalanine, tyrosine, dopamine, adrenaline/noradrenaline

**Figure 6:**
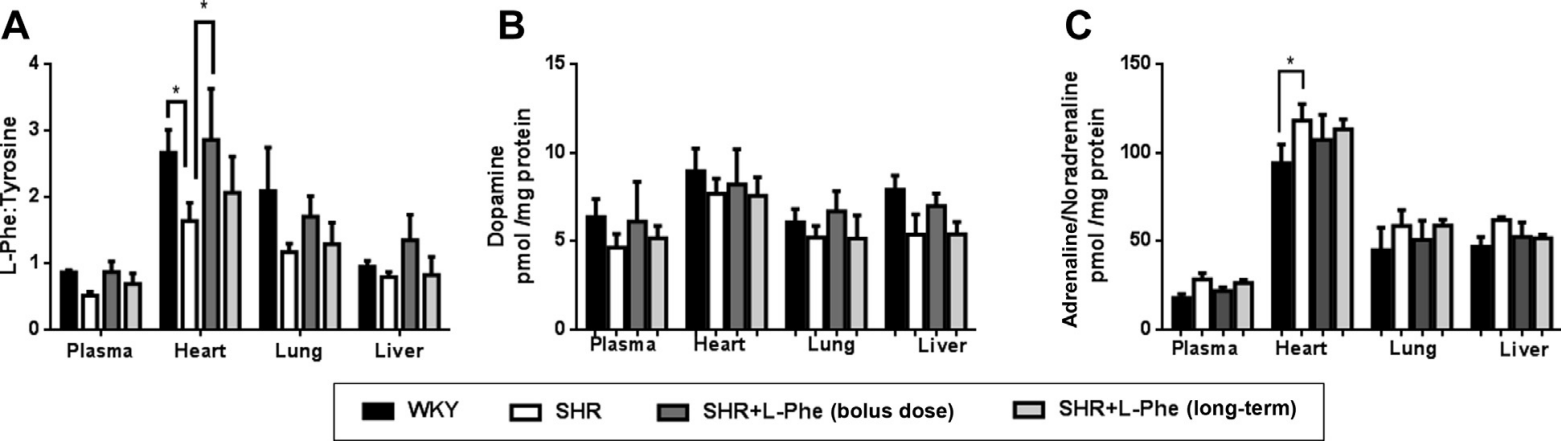
Effects of Short-Term and Long-Term L-Phe on Systemic and Tissue Phenylalanine, Tyrosine, Dopamine, and Adrenaline/Noradrenaline Effects of short-term (100 mg·kg^−1^ L-phe, oral gavage, 4 h, n = 6) and long-term administration (2.5% L-phe in drinking water for 60 days, n = 6) in SHR and WKY on levels of different catecholamines: **(A)** L-phe/L-tyr ratio, **(B)** dopamine, and **(C)** adrenaline/noradrenaline in plasma and tissues. The datasets shown represent the mean ± SEM (n = 6) where *p < 0.05 and **p < 0.01. Abbreviations as in Figure 1.

L-phe is metabolized to l-tyrosine (L-tyr) via the action of phenylalanine hydroxylase in vivo. The ratio of L-phe/L-tyr was higher in WKY compared with SHR basally. However, the L-phe/L-tyr ratio was increased following a bolus dose of L-phe challenge in SHR, confirming that L-phe was absorbed following oral gavage (Figure 6A, Supplemental Table 1). We did not detect a significant rise in the L-phe/L-tyr ratio in animals treated long-term with L-phe. This is not surprising because the long-term ad libitum L-phe dosing was at a much lower dose than the short-term bolus dose challenge. Although there were trends of decreased dopamine and increased adrenaline/noradrenaline between SHR and WKY, these did not reach statistical significance in most tissues. The exception was the heart, where adrenaline/noradrenaline levels were significantly higher in SHR than WKY basally, but equaled WKY levels following L-phe treatment (Figures 6B and 6C).

## Discussion

The salient findings of this work are that oral supplementation of the dietary amino acid L-phe was able to enhance endogenous BH_4_ biosynthesis through the GCH1-GFRP protein complex, elevate nitrite levels, reduce vascular ROS levels, and improve endothelium-dependent vascular relaxation. The functional improvements of L-phe were seen only in hypertensive animals (SHR), and no change in functional reactivity was observed in normotensive (WKY) controls. These beneficial effects were achieved following both high bolus dose short-term challenge and following long-term lower-dose ad libitum supplementation in the drinking water. BH_2_ and catecholamine levels were not significantly altered by L-phe.

Importantly, this is a proof-of-concept study demonstrating that GCH1-GFRP is a rational therapeutic target for vascular dysfunction. Hence, the development of L-phe mimetics that selectively bind to and enhance this protein complex may be of clinical value. Our data suggest that L-phe could itself be translated into the clinic given the minimal effects observed on catecholamines but should be advanced with caution, given L-phe’s diverse biological action and potential for predictable adverse drug reactions.

GCH1 binds to GFRP to form a protein complex that is receptive to allosteric regulation by both L-phe (feed forward) and BH_4_ (feedback) regulation (21). Our results have confirmed numerous previous reports that L-phe only enhances GCH1 activity when it is bound to GFRP 24, 30.

In addition to its essential cofactor role for NOS activation, BH_4_ is also required by phenylalanine hydroxylase to catalyze the conversion of L-phe to L-tyr, which is further converted to dopamine, adrenaline, and noradrenaline (41). L-phe thus regulates its own metabolism via feed-forward activation of GCH1-GFRP with subsequent increases in BH_4_ and hence phenylalanine hydroxylase activity. This is important because sustained elevation of L-phe can become neurotoxic (42). Indeed, BH_4_ has been successfully used as a treatment for a subset of patients with phenylketonuria (43). Consistent with raised biopterin levels seen in patients after L-phe loading (23) and our previous observations in mice (24), plasma BH_4_ levels were significantly increased in control WKY and SHR after 100 mg·kg^−1^ L-phe bolus oral challenge in the present study.

The SHR is an appropriate model to study endothelial dysfunction because the animals demonstrate reduced NO signaling, reduced endothelial-dependent vascular relaxation, enhanced cardiovascular remodeling, and increased oxidative stress 19, 44, 45. In this study, lung BH_4_ levels were lower in SHR than in age-matched WKY, consistent with published reports (46). Following a short-term oral dose (4 h) or long-term daily (8 weeks) L-phe challenge, tissue BH_4_ levels in SHR were restored to control WKY levels.

Correspondingly, we observed increased aortic superoxide production in SHR basally and L-phe administration increased aortic BH_4_ and concomitantly reduced superoxide levels. These data support the hypothesis that L-phe activates the GCH1-GFRP complex in vivo, raising endogenous BH_4_ biosynthesis to support full “coupled” NOS activity, thereby reducing oxidative stress in this model of hypertension.

Limited BH_4_ bioavailability is believed to lead to NOS uncoupling, generating superoxide instead of NO 8, 47. In SHR, the observed endothelial dysfunction is, in part, a result of eNOS uncoupling attributed to BH_4_ insufficiency and NO scavenging by reactive oxygen species (ROS) 48, 49, 50. Interestingly, in this study, L-phe caused a more sustained increase of plasma nitrite in SHR compared with WKY. This unexpected, but welcome, observation may be explained by an exaggerated improvement from a short-term surge in BH_4_ in the already compromised SHR. In other words, the spike in BH_4_ following L-phe dosing may have temporarily “recoupled” NOS and elicited further direct antioxidant effects on other ROS, thereby greatly enhancing NO and hence nitrite levels. By contrast, WKY should not have BH_4_ insufficiency and should have “fully coupled” eNOS. Hence, the short-term effects of BH_4_ elevation on nitrite should be less pronounced. Further mechanistic studies would be required to confirm this suggestion.

To verify whether L-phe could activate the GCH1-GFRP axis functionally, a series of studies were carried out using fresh conduit (aortic) and resistance (mesenteric) blood vessels from WKY and SHR. Consistent with published reports, Ach-mediated vascular relaxation in SHR was significantly impaired in comparison to WKY rats. Short-term L-phe incubation within the organ bath significantly improved vascular relaxation in SHR vessels yet had no effect on WKY. This implies that L-phe, via local elevation of BH_4_ within the vasculature, enhances NO bioavailability and endothelial function only in circumstances where the pathway is dysfunctional. This is consistent with the differential effects on plasma nitrite between SHR and WKY discussed in the preceding text. Interestingly, L-phe had a more pronounced effect on vascular relaxation in mesenteric arteries, suggesting that non-NO–mediated pathways may have likely been involved as well. Indeed, within this vascular bed, endothelium-derived hyperpolarizing factor (EDHF) has been proposed to play a prominent role 51, 52, 53. Hydrogen peroxide has been shown to induce EDHF-like relaxations and promotes endothelium-dependent and -independent relaxations 52, 54, 55. BH_4_ can react with molecular oxygen producing hydrogen peroxide, thereby offering an additional pathway to promote vascular relaxation within such resistance vessels 10, 56. Thus, the improved Ach-induced relaxations by L-phe in mesenteric arteries may have also been mediated by an improvement of EDHF-like signaling. To rule out the influence of prostaglandins on vascular relaxation, indomethacin was employed throughout these studies.

BH_4_ is highly unstable and can be rapidly oxidized to BH_2_. Previously, studies have demonstrated that BH_2_ can itself bind to the BH_4_ binding site on NOS but does not confer any cofactor functionality. Thus, raised BH_2_ can be problematic, competing with BH_4_ for the NOS binding site and promoting NOS uncoupling. Importantly, in our study, we did not observe any significant differences in BH_2_ levels following L-phe supplementation. This was a surprising finding because elevated BH_4_ is typically associated with a corresponding rise in BH_2_. Whether the absence of BH_2_ elevation was a consequence of our experimental design and the time points investigated—or a potential antioxidant effect of L-phe—remains to be determined.

It would now be important to establish the in vivo significance of our findings and to ascertain whether activation of the GCH1-GFRP axis could attenuate the development of hypertension in vivo in SHR and in the clinic. We have previously postulated that L-phe is not necessarily a viable therapeutic agent in itself, given its role in catecholamine biosynthesis. However, our data revealed no significant effects following both short-term and long-term L-phe challenge on dopamine, noradrenaline, and adrenaline levels, indicating that endogenous regulatory mechanisms may tightly control the bioavailability of these catecholamines. However, our study used L-phe supplementation over a relatively short period of time and does not reflect the projected timeframe for clinical therapeutics (years). Thus, the observed lack of effect of L-phe on catecholamine biosynthesis should be treated with caution and further investigated to fully understand the impact on non–GCH1-BH_4_-NO pathways. Interestingly, SHR display significantly lower basal L-phe levels compared with WKY; this was not anticipated and may suggest that the bioavailability of L-phe may influence the GCH1-GFRP complex and hence vascular regulation in these animals.

Our study builds upon an existing published reports where numerous attempts to restore NO bioavailability have been trialed by targeting different elements of the NOS-NO pathway. These pathways include substrate enhancement (via l-arginine supplementation), cofactor enhancement (via BH_4_ supplementation), or manipulation of the endogenous NOS inhibitors (asymmetric dimethylarginine [ADMA] and l-NMMA) (57). l-Arginine supplementation is by far the most extensively studied, but results have been variable. Indeed the VINTAGE MI (Vascular Interaction With Age in Myocardial Infarction) randomized controlled trial demonstrated not only lack of efficacy but also higher post-infarction mortality (58). Further, a meta-analysis of l-arginine supplementation in myocardial infarction showed no efficacy (59). Thus, there are efficacy and safety concerns regarding l-arginine supplementation in cardiovascular disease patients. The endogenous NOS inhibitor, ADMA competes with l-arginine for the substrate binding site of NOS, and hence the efficacy of supplemented l-arginine is dependent upon the intracellular arginine/ADMA ratio. Finally, l-arginine has poor oral bioavailability (60), likely attributable to intestinal arginase activity, and hence l-citrulline has more recently been suggested as an alternative method to enhance l-arginine bioavailability (61).

Thus, there is still much work to be undertaken to improve the efficacy and safety of pharmacotherapies that enhance NO bioavailability, but our present study provides the first proof-of-concept data that the GCH1-GFRP complex is a rational therapeutic target to achieve BH_4_ elevation and NO restoration within blood vessels.

To translate these findings further, we propose 2 parallel research strategies. The first clinical development strategy would investigate the impact of L-phe administration on vascular function using flow mediated dilatation, in patients with existing endothelial dysfunction versus healthy controls. It may be predicted that flow-mediated dilatation would be improved in the patient cohort, whereas negligible effect would be seen in the nonpatient controls. However, L-phe, as a therapy, may have challenges given its diverse biological activity, raising potential safety concerns, and these would need to be concomitantly investigated in trial participants. The second parallel strategy would be focused around drug discovery, to identify small molecules that selectively bind to and activate the GCH1-GFRP complex without displaying the dual substrate activity for phenylalanine hydroxylase, minimizing the potential for off-target adverse drug reactions.

## Conclusions

Our proof-of-concept study confirms that activation of GCH1-GFRP can directly affect vascular BH_4_, NO, and ROS and restore vascular function in a model of hypertension. This was achieved using the dietary amino acid L-phe. It now remains to be determined whether small-molecule L-phe mimetics require development or if L-phe itself is a safe and efficacious treatment for endothelial dysfunction.

Perspectives **COMPETENCY IN MEDICAL KNOWLEDGE:** L-phe–mediated GCH1-GFRP activation leads to a rise in vascular BH_4_ levels and improved vascular relaxation in a rodent model of hypertension. Small molecules that mimic this allosteric activation of GCH1 represent a potential novel therapy to treat a diverse range of cardiovascular diseases underpinned by limited NO and/or enhanced oxidative stress.

**TRANSLATIONAL OUTLOOK:** Our studies demonstrate the mechanism via which L-phe restores endothelial function in a model of hypertension, indicating that the GCH1-GFRP complex represents a viable therapeutic target for the restoration of endothelial function. This method circumvents the potential oxidative inactivation of BH_4_ following oral dosing. Although L-phe has been used as a tool to probe the GCH1-GFRP pathway, it may not be a viable therapeutic agent, given its precursor role for the biosynthesis of catecholamines. However, small molecules that mimic the allosteric effects of L-phe at the GCH1-GFRP interface but do not bind to phenylalanine hydroxylase could be developed, underpinned by endothelial dysfunction.

## References

[1] Heidenreich P.A., Trogdon J.G., Khavjou O.A. (2011). Forecasting the future of cardiovascular disease in the United States: a policy statement from the American Heart Association. Circulation.

[2] Briones A.M., Touyz R.M. (2010). Oxidative stress and hypertension: current concepts. Curr Hypertens Rep.

[3] Endemann D.H., Schiffrin E.L. (2004). Nitric oxide, oxidative excess, and vascular complications of diabetes mellitus. Curr Hypertens Rep.

[4] Davignon J., Ganz P. (2004). Role of endothelial dysfunction in atherosclerosis. Circulation.

[5] Forstermann U., Sessa W.C. (2012). Nitric oxide synthases: regulation and function. Eur Heart J.

[6] Kwon N.S., Nathan C.F., Stuehr D.J. (1989). Reduced biopterin as a cofactor in the generation of nitrogen oxides by murine macrophages. J Biol Chem.

[7] Tayeh M.A., Marletta M.A. (1989). Macrophage oxidation of L-arginine to nitric oxide, nitrite, and nitrate- tetrahydrobiopterin is required as a cofactor. J Biol Chem.

[8] Li L., Chen W., Rezvan A., Jo H., Harrison D.G. (2011). Tetrahydrobiopterin deficiency and nitric oxide synthase uncoupling contribute to atherosclerosis induced by disturbed flow. Arterioscler Thromb Vasc Biol.

[9] Crabtree M.J., Channon K.M. (2011). Synthesis and recycling of tetrahydrobiopterin in endothelial function and vascular disease. Nitric Oxide.

[10] Starr A., Hussein D., Nandi M. (2013). The regulation of vascular tetrahydrobiopterin bioavailability. Vasc Pharmacol.

[11] Porkert M., Sher S., Reddy U. (2008). Tetrahydrobiopterin: a novel antihypertensive therapy. J Hum Hypertens.

[12] Maier W., Cosentino F., Lutolf R.B. (2000). Tetrahydrobiopterin improves endothelial function in patients with coronary artery disease. J Cardiovasc Pharmacol.

[13] Cosentino F., Huerlimann D., Gatti C.D. (2008). Chronic treatment with tetrahydrobiopterin reverses endothelial dysfunction and oxidative stress in hypercholesterolaemia. Heart.

[14] Cunnington C., Van Assche T., Shirodaria C. (2012). Systemic and vascular oxidation limits the efficacy of oral tetrahydrobiopterin treatment in patients with coronary artery disease. Circulation.

[15] Reverter E., Mesonero F., Seijo S. (2015). Effects of sapropterin on portal and systemic hemodynamics in patients with cirrhosis and portal hypertension: a bicentric double-blind placebo-controlled study. Am J Gasterenterol.

[16] De Maria R., Campolo J., Frontali M. (2014). Effects of sapropterin on endothelium-dependent vasodilation in patients with CADASIL a randomized controlled trial. Stroke.

[17] Nichol C.A., Lee C.L., Edelstein M.P., Chao J.Y., Duch D.S. (1983). Biosynthesis of tetrahydrobiopterin by denovo and salvage pathways in adrenal medulla extracts, mammalian-cell cultures and rat brain in vivo. Proc Natl Acad Sci U S A.

[18] Carnicer R., Hale A.B., Suffredini S. (2012). Cardiomyocyte GTP cyclohydrolase 1 and tetrahydrobiopterin increase NOS1 activity and accelerate myocardial relaxation. Circ Res.

[19] Cosentino F., Luscher T.F. (1998). Tetrahydrobiopterin and endothelial function. Eur Heart J.

[20] Chuaiphichai S., McNeill E., Douglas G. (2014). Cell-autonomous role of endothelial GTP cyclohydrolase 1 and tetrahydrobiopterin in blood pressure regulation. Hypertension.

[21] Harada T., Kagamiyama H., Hatakeyama K. (1993). Feedback regulation mechanisms for the control of GTP cyclohydrolase-1 activity. Science.

[22] Yoneyama T., Hatakeyama K. (2001). Ligand binding to the inhibitory and stimulatory GTP cyclohydrolase I/GTP cyclohydrolase I feedback regulatory protein complexes. Protein Sci.

[23] Saunders-Pullman R., Blau N., Hyland K. (2004). Phenylalanine loading as a diagnostic test for DRD: interpreting the utility of the test. Mol Genet Metab.

[24] Hussein D., Starr A., Heikal L., Nandi M. (2015). Investigating the interaction between GTP-cyclohydrolase1 and its feedback regulatory protein. Nitric Oxide.

[25] Li L., Rezvan A., Salerno J.C. (2010). GTP cyclohydrolase I phosphorylation and interaction with GTP cyclohydrolase feedback regulatory protein provide novel regulation of endothelial tetrahydrobiopterin and nitric oxide. Circ Res.

[26] Kalivendi S., Hatakeyama K., Whitsett J., Konorev E., Kalyanaraman B., Vasquez-Vivar J. (2005). Changes in tetrahydrobiopterin levels in endothelial cells and adult cardiomyocytes induced by LPS and hydrogen peroxide: a role for GFRP?. Free Radic Biol Med.

[27] Nandi M., Kelly P., Vallance P., Leiper J. (2008). Over-expression of GTP-cyclohydrolase 1 feedback regulatory protein attenuates LPS and cytokine-stimulated nitric oxide production. Vasc Med.

[28] d'Uscio L.V., Katusic Z.S. (2006). Increased vascular biosynthesis of tetrahydrobiopterin in apolipoprotein E- deficient mice. Am J Physiol Heart Circ Physiol.

[29] Gesierich A., Niroomand F., Tiefenbacher C.P. (2003). Role of human GTP cyclohydrolase I and its regulatory protein in tetrahydrobiopterin metabolism. Basic Res Cardiol.

[30] Kolinsky M.A., Gross S.S. (2004). The mechanism of potent GTP cyclohydrolase I inhibition by 2,4-diamino-6-hydroxypyrimidine: requirement of the GTP cyclohydrolase I feedback regulatory protein. J Biol Chem.

[31] Kilkenny C., Browne W.J., Cuthill I.C., Emerson M., Altman D.G. (2010). Improving bioscience research reporting: the ARRIVE guidelines for reporting animal research. PloS Biol.

[32] Howells D.W., Smith I., Hyland K. (1986). Estimation of tetrahydrobiopterin and other pterins in cerebrospinal fluid using reversed phase high performance liquid chromatography with electrochemical and fluorescence detection. J Chromatogr.

[33] Starr A., Sand C.A., Heikal L. (2014). Overexpression of GTP cyclohydrolase 1 feedback regulatory proteins is protective in a murine model of septic shock. Shock.

[34] Cai S., Alp N.J., McDonald D. (2002). GTP cyclohydrolase I gene transfer augments intracellular tetrahydrobiopterin in human endothelial cells: effects on nitric oxide synthase activity, protein levels and dimerisation. Cardiovasc Res.

[35] Verdon C.P., Burton B.A., Prior R.L. (1995). Sample pretreatment with nitrate reductase and glucose-6-phosphate-dehydrogenase quantitatively reduces nitrate while avoiding interference by NADP(+) when the Griess reaction is used to assay for nitrite. Analytical Biochemistry.

[36] Bryan N.S., Grisham M.B. (2007). Methods to detect nitric oxide and its metabolites in biological samples. Free Radic Biol Med.

[37] Li J.M., Shah A.M. (2001). Differential NADPH- versus NADH-dependent superoxide production by phagocyte-type endothelial cell NADPH oxidase. Cardiovasc Res.

[38] Atherton N.D., Green A. (1988). HPLC measurement of phenylalanine in plasma. Clin Chem.

[39] Heikal L., Aaronson P.I., Ferro A., Nandi M., Martin G.P., Dailey L.A. (2011). S- Nitrosophytochelatins: investigation of the bioactivity of an oligopeptide nitric oxide delivery system. Biomacromolecules.

[40] Mulvany M.J., Halpern W. (1977). Contractile properties of small arterial resistance vessels in spontaneously hypertensive and normotensive rats. Circ Res.

[41] Kaufman S. (1958). New cofactor required for the enzymatic conversion of phenylalanine to tyrosine. J Biol Chem.

[42] Heintz C., Cotton R.G.H., Blau N. (2013). Tetrahydrobiopterin, its mode of action on phenylalanine hydroxylase, and importance of genotypes for pharmacological therapy of phenylketonuria. Hum Mutat.

[43] Scala I., Concolino D., Della Casa R. (2015). Long-term follow-up of patients with phenylketonuria treated with tetrahydrobiopterin: a seven years experience. Orphanet J Rare Dis.

[44] Aboudonia M.M., Duch D.S., Nichol C.A., Viveros O.H. (1983). Hormonal regulation of guanosine triphosphate cyclohydrolase activity and biopterin levels in the rat adrenal cortex. Endocrinology.

[45] Bernatova I., Conde M.V., Kopincova J., Gonzalez M.C., Puzserova A., Arribas S.M. (2009). Endothelial dysfunction in spontaneously hypertensive rats: focus on methodological aspects. J Hypertens Suppl.

[46] Hong H.J., Hsiao G., Cheng T.H., Yen M.H. (2001). Supplementation with tetrahydrobiopterin suppresses the development of hypertension in spontaneously hypertensive rats. Hypertension.

[47] Alp N.J., McAteer M.A., Khoo J., Choudhury R.P., Channon K.M. (2004). Increased endothelial tetrahydrobiopterin synthesis by targeted transgenic GTP-cyclohydrolase I overexpression reduces endothelial dysfunction and atherosclerosis in apoE-knockout mice. Arterioscler Thromb Vasc Biol.

[48] Bhatt S.R., Lokhandwala M.F., Banday A.A. (2011). Resveratrol prevents endothelial nitric oxide synthase uncoupling and attenuates development of hypertension in spontaneously hypertensive rats. Eur J Pharmacol.

[49] Li H.G., Witte K., August M. (2006). Reversal of endothelial nitric oxide synthase uncoupling and up-regulation of endothelial nitric oxide synthase expression lowers blood pressure in hypertensive rats. J Am Coll Cardiol.

[50] Landmesser U., Dikalov S., Price S.R. (2003). Oxidation of tetrahydrobiopterin leads to uncoupling of endothelial cell nitric oxide synthase in hypertension. J Clin Invest.

[51] Archer S.L., Gragasin F.S., Wu X.C. (2003). Endothelium-derived hyperpolarizing factor in human internal mammary artery is 11,12-epoxyeicosatrienoic acid and causes relaxation by activating smooth muscle BKCa channels. Circulation.

[52] Ozkor M.A., Quyyumi A.A. (2011). Endothelium-derived hyperpolarizing factor and vascular function. Cardiol Res Pract.

[53] Scotland R.S., Madhani M., Chauhan S. (2005). Investigation of vascular responses in endothelial nitric oxide synthase/cyclooxygenase-1 double-knockout mice: key role for endothelium-derived hyperpolarizing factor in the regulation of blood pressure in vivo. Circulation.

[54] Rubanyi G.M., Romero J.C., Vanhoutte P.M. (1986). Flow-induced release of endothelium-derived relaxing factor. Am J Physiol.

[55] Chidgey J., Fraser P.A., Aaronson P.I. (2016). Reactive oxygen species facilitate the EDH response in arterioles by potentiating intracellular endothelial Ca2+ release. Free Radic Biol Med.

[56] Garry A., Edwards D.H., Fallis I.F., Jenkins R.L., Griffith T.M. (2009). Ascorbic acid and tetrahydrobiopterin potentiate the EDHF phenomenon by generating hydrogen peroxide. Cardioavasc.

[57] Rochette L., Lorin J., Zeller M. (2013). Nitric oxide synthase inhibition and oxidative stress in cardiovascular diseases: possible therapeutic targets?. Pharmacol Ther.

[58] Schulman S.P., Becker L.C., Kass D.A. (2006). L-arginine therapy in short-term myocardial infarction: the Vascular Interaction With Age in Myocardial Infarction (VINTAGE MI) randomized clinical trial. JAMA.

[59] Sun T., Zhou W.B., Luo X.P., Tang Y.L., Shi H.M. (2009). Oral L-arginine supplementation in acute myocardial infarction therapy: a meta-analysis of randomized controlled trials. Clinical Cardiology.

[60] Schwedhelm E., Maas R., Freese R. (2008). Pharmacokinetic and pharmacodynamic properties of oral L-citrulline and L-arginine: impact on nitric oxide metabolism. Br J Clin Pharmacol.

[61] Morita M., Hayashi T., Ochiai M. (2014). Oral supplementation with a combination of L-citrulline and L-arginine rapidly increases plasma L-arginine concentration and enhances NO bioavailability. Biochem Biophys Res Commun.

