# Supplementary material for: l-Phenylalanine Restores Vascular Function in Spontaneously Hypertensive Rats Through Activation of the GCH1-GFRP Complex
Source: JACC Basic Transl Sci. 2018 May 30;3(3):366–77. doi: 10.1016/j.jacbts.2018.01.015 (PMC6018612; doi:10.1016/j.jacbts.2018.01.015)
Supplement: Supplemental Data [file mmc1.pdf]

SUPPLEMENTAL METHODS

Effect of L-phe on GCH-1 recombinant protein activity

A kinetic microplate assay was used to determine GCH1 activity over time [1], in which substrate GTP (100µmol/L, Fermentas,), GFRP (1µmol/L) and L-phe (1 mmol/L, Alfa Aesar, UK) was added to purified GCH1 protein (0.1 µmol.L^{-1}). The generation of the intermediate reaction product, dihydroneopterin triphosphate (DHNTP) was quantified using a Spectramax temperature controlled plate reader as previously described [2]. Absorbance units were expressed in molar concentration of DHNTP where the extinction coefficient of DHNTP ε_{340}=0.091 M^{-1} cm^{-1}.

Calculations of L-phe to be added to drinking water for chronic treatment of SHR:

High L-phe diet is considered to be =4-7% per day [3]
Percentage of Phe in rodent chow is 0.66%
Based on our own observation and published literature [4, 5], the daily food intake of adult rats is ~ 20 g
*20 g daily intake = **0.132g** L-phe from solid diet per day*

2.5% maximum solubility of L-phe in water. Total daily water intake 25 ml per day [6]
2.5g L-phe in 100ml = 2.5% w/v
*25ml daily consumption = **0.625g** L-phe from water per day.*
*Total daily consumption of L-phe = 0.132+0.625 = **0.757g***

Therefore, for chronic studies, total L-phe intake:
*L-phe supplemented rats receive **0.757g per day = 4% (high L-phe)***
*Un-supplemented control receive **0.132 g per day = 0.66% (standard L-phe)***

Animals

Experiments were conducted at approximately the same time of day to account for diurnal variation. Drug administration was randomised and un-blinded, but data was analysed by a blinded investigator. **Historical data from our laboratory and previous published reports have shown that SHR become hypertensive after 5-7 weeks of age, when compared to controls, and the studies described in the main methods therefore incorporate this time frame (Online Fig 1)** [7-9].

Two independent studies were conducted to investigate the impact of L-phe administration on vascular reactivity. The first focused on the effects of short-term bolus L-phe dosing in established hypertension using 13 week old SHR. The second investigated the impact of long-term L-phe supplementation, in pre-hypertensive animals from 4-5 weeks of age. Thus, 3 week old or 12 week old SHR and WKY were purchased from a commercial supplier (Envigo, UK) and rats were acclimatized in home cages for 1 week prior to experimentation with *ad libitum* access to food and water and on a 12 hour light-dark cycle. **Routes of drug administration, surgical protocols and data analysis are detailed in the main methods section.**

Sample preparation for measurement of analytes

For all studies, rats were humanely killed by terminal bleed under anaesthesia (2% inhaled isoflurane). Venous blood was collected into heparinised tubes (100 $U.mL^{-1}$) and plasma was separated by centrifugation and snap frozen in liquid nitrogen. Similarly, dissected identical sections of aorta, heart, liver, and lung tissues were snap frozen, whole. The remainder of these tissues were homogenised in 100mg tissue/ml phosphate buffered saline (PBS; pH 7.4). Plasma, whole tissue and homogenates were stored at -80 degrees centigrade until required for BH_4, phenylalanine, tyrosine, dopamine, adrenaline/noradrenaline and nitrite measurement.

Finally, fresh aorta and mesenteric artery was carefully dissected and placed in cold Krebs' buffer for the measurement of reactive oxygen species and functional vascular reactivity.

Nitrite measurement

Quantification of total NOx was performed as previously described [10]. Briefly, plasma and tissue samples were de-proteinated and quantified using a fluorometric method utilizing 2,3-diaminonaphthalene [11]. Nitrite was expressed as $\mu mol.L^{-1}$ /mg protein following quantification of protein concentration using the Bradford assay.

Quantification of superoxide levels

Superoxide levels were quantified using a lucigenin chemiluminescence-based assay as previously described [12]. Briefly, aortic rings (2mm) were suspended in cold 100 µL Krebs solution (119 $mmol.L^{-1}$ NaCl, 4.7 $mmol.L^{-1}$ KCl, 1.5 $mmol.L^{-1}$ $MgSO_4$, 2.5 $mmol.L^{-1}$ $CaCl_2$, 25 $mmol.L^{-1}$ $NaHCO_3$, 1.2 $mmol.L^{-1}$ KH_2PO_4, 11 $mmol.L^{-1}$ glucose and 100 $\mu mol.L^{-1}$ L-arginine, pH 7.4) in a 96-well microplate luminometer (Model Lucy 1, Rosys Anthos, Austria). NADPH (100 $\mu mol.L^{-1}$) and lucigenin; bis-*N*-methylacridinium nitrate (10 $\mu mol.L^{-1}$) was added prior to measurement, in the presence or absence of superoxide dismutase (SOD, 200 $units.mL^{-1}$). Light emission was recorded as mean arbitrary light units/cycle over 80 cycles.

Aromatic amino acid and catecholamine measurement

Phenylalanine, tyrosine, dopamine and adrenaline/noradrenaline were measured by UV spectrophotometric detection in plasma and tissues [13]. Briefly, tissue homogenate (PBS) or plasma were de-proteinated with an equal volume of perchloric acid solution (PCA, 0.59 $mol.L^{-1}$). The clear supernatant was then used to separate different analytes by HPLC (Jasco Plus, UK) and quantified using UV spectrophotometric detection (wavelength 210 nm). HPLC separation was performed using a Aeris PEPTIDE C18 column (3.6u, 150 x 4.6mm, Phenomenex), and a mobile phase comprising Acetonitrile (2.5%), Octylamine (10 $\mu L.L^{-1}$) and Perchloric acid (0.8 $mL.L^{-1}$), pH 2, at a flow rate of 0.75ml/min. Quantitation of dopamine, L-phenylalanine, L-tyrosine and adrenaline/noradrenaline was performed by comparison with external standards (0-250 $mmol.L^{-1}$) and normalized for total protein. The ratio of L-phe: tyrosine was further calculated. The lower limits of detection were 10.3, 2.7, 4.5 and 41.1 $umol.L^{-1}$ for dopamine, L-phenylalanine, L-tyrosine and adrenaline/noradrenaline, respectively.

REFERENCES

1. Kolinsky, M.A. and S.S. Gross, *The mechanism of potent GTP cyclohydrolase I inhibition by 2,4-diamino-6-hydroxypyrimidine - Requirement of the GTP cyclohydrolase I feedback regulatory protein.* Journal of Biological Chemistry, 2004. **279**(39): p. 40677-40682.
2. Hussein, D., et al., *Investigating the interaction between GTP-cyclohydrolase1 and its feedback regulatory protein.* Nitric Oxide, 2015. **27, Supplement**(0): p. S29-S30.
3. Iijima, S., et al., *Studies on experimental phenylketonuria in rats.* Tohoku Journal of Experimental Medicine, 1975. **117**(2): p. 167-178.
4. Vento, P.J., et al., *Food Intake in Laboratory Rats Provided Standard and Fenbendazole-supplemented Diets.* Journal of the American Association for Laboratory Animal Science, 2008. **47**(6): p. 46-50.
5. *http://www.researchdiets.com/resource-center-page/typical-food-intake*. Available from: http://www.researchdiets.com/resource-center-page/typical-food-intake.
6. McGivern, R.F., et al., *Sex difference in daily water consumption of rats: Effect of housing and hormones.* Physiology & Behavior, 1996. **59**(4-5): p. 653-658.
7. Okamoto, K. and K. Aoki, *Development of a strain of spontaneously hypertensive rats.* Japan Circulation Jour, 1963. **27**((3)): p. 282-293.
8. Hom, S., et al., *Comparative changes in the blood-brain barrier and cerebral infarction of SHR and WKY rats.* American Journal of Physiology-Regulatory Integrative and Comparative Physiology, 2007. **292**(5): p. R1881-R1892.
9. Dickhout, J.G. and R. Lee, *Blood pressure and heart rate development in young spontaneously hypertensive rats.* American Journal of Physiology-Heart and Circulatory Physiology, 1998. **274**(3): p. H794-H800.
10. Verdon, C.P., B.A. Burton, and R.L. Prior, *Sample pretreatment with nitrate reductase and glucose-6-phosphate-dehydrogenase quantitatively reduces nitrate while avoiding interference by NADP(+) when the Griess reaction is used to assay for nitrite.* Analytical Biochemistry, 1995. **224**(2): p. 502-508.
11. Bryan, N.S. and M.B. Grisham, *Methods to detect nitric oxide and its metabolites in biological samples.* Free Radical Biology and Medicine, 2007. **43**(5): p. 645-657.
12. Li, J.M. and A.M. Shah, *Differential NADPH- versus NADH-dependent superoxide production by phagocyte-type endothelial cell NADPH oxidase.* Cardiovascular Research, 2001. **52**(3): p. 477-486.
13. Atherton, N.D. and A. Green, *HPLC measurement of phenylalanine in plasma.* Clinical Chemistry, 1988. **34**(11): p. 2241-2244.

Supplemental Figure 1

Radiotelemetry data showing blood pressure profile in conscious unrestrained rats (systolic, diastolic and mean arterial blood pressure) expressed in mmHg and development of hypertension in SHR at 6 weeks age (pre-hypertensive age) over a period of 8 weeks.

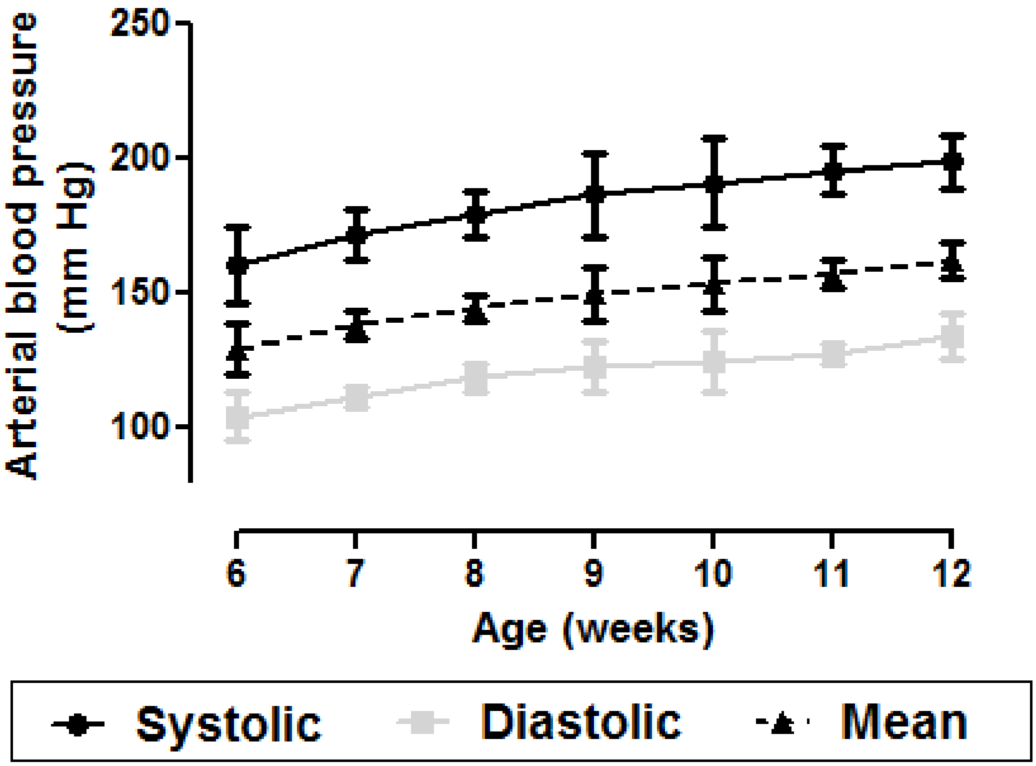

Supplemental Figure 2

Effects of acute (100 mg.kg-1 L-phe, oral gavage, 4h, n=6) and chronic administration (2% L-phe in drinking water for 60 days, n=6) on levels of nitrite and BH_4, BH2 and biopterin in plasma. The data sets shown represent the mean ± SEM. In each data set p value was >0.05 (no significant difference).

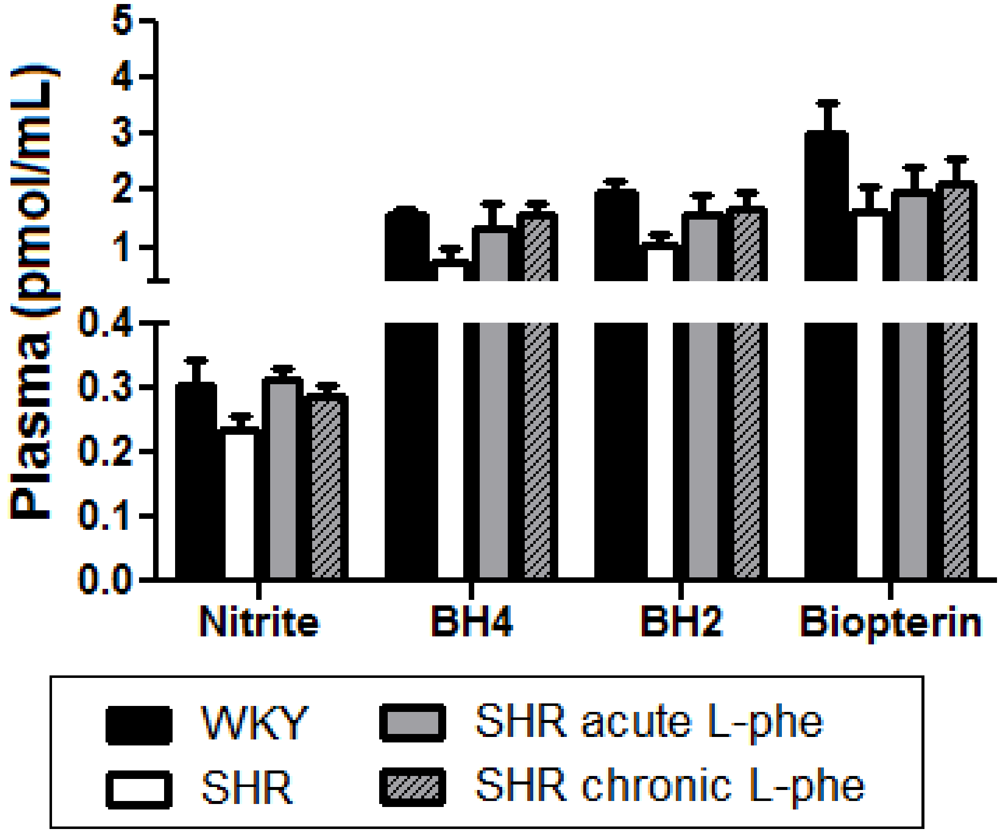

Supplemental Table 1

Effects of acute (100 mg.kg-1 L-phe, oral gavage, 4h, n=6) and chronic administration (2% L-phe in drinking water for 60 days, n=6) in SHRs and WKY rats on levels of L-phe and L-tyrosine The data sets shown represent the mean ± SEM (n=6).

	L-phe (pmol.mg^{-1} protein)				L-tyrosine (pmol.mg^{-1} protein)			
	WKY	SHR	+L-Phe (acute)	+ L-Phe (chronic)	WKY	SHR	+L-Phe (acute)	+ L-Phe (chronic)
**Plasma**	7.8±1.4	6.9±1.9	7.6±1.4	6.9±1.6	9.2±2.9	12.2±5.1	9.8±2.1	11.2±3.6
**Heart**	9.3±3.5	8.1±1.1	9.7±2.8	8.5±2.8	5.1±1.2	4.2±1.6	4.4±1.9	5.3±1.2
**Lung**	12.8±2.4	11.5±2.4	14.3±1.9	11.5±2.5	8.6±3.7	10.7±3.1	9.4±2.7	9.9±3.5
**Liver**	10.2±3.4	10.7±3.1	10.6±3.1	11.09±3.6	11.3±4.7	13.4±2.7	12.3±3.8	17.9±7.5

Supplemental Table 2

Tabulated p-values: When not able to be detailed within the main results section, exact p values have been tabulated.

Figure 1a	p-values
GCH1+GFRP vs GCH1 alone	0.009
GCH1+GFRP +L-Phe vs GCH1 alone	0.0007

Figure 1b	BH_4 treatment	p-values
WKY	0.5 h vs 0 h	<0.0001
	1 h vs 0 h	0.036
SHR	0.5 h vs 0 h	0.0065
	1 h vs 0 h	0.012

Figure 1c	Nitrite treatment	p-values
WKY	0.5 h vs 0 h	0.0096
SHR	0.5 h vs 0 h	0.012
	1 h vs 0 h	0.0038
	4 h vs 0 h	0.0056

Figure 3	Groups	p value
3a. Aortic BH4	WKY vs SHR	0.034
	SHR vs chronic L-Phe treated	0.018
3b. Aortic superoxide	WKY vs SHR (- SOD)	<0.001
	SHR (-SOD) vs SHR (+SOD)	<0.001
	Acute L-phe (-SOD) vs (+SOD)	0.0043
	Chronic L-Phe (-SOD) vs (+SOD)	0.0021
	SHR (-SOD) vs acute L-Phe(-SOD)	0.024
	SHR (-SOD) vs chronic L-Phe (-SOD)	0.039

Figure 2	Tissue	Groups	p value
2a. BH_4	Lung	WKY vs SHR	<0.001
		SHR vs L-Phe treated	0.033
	Liver	SHR vs acute L-Phe treated	0.027
		SHR vs chronic L-Phe treated	0.019
2b. Nitrite	Heart	WKY vs SHR	0.0067
	Lung	WKY vs SHR	<0.001
		SHR vs chronic L-Phe treated	0.041
	Liver	WKY vs SHR	0.027
		SHR vs acute L-Phe treated	<0.001
2c. BH_2	Lung	WKY vs SHR	0.041
	Liver	WKY vs SHR	0.0075
2d. Biopterin	Lung	WKY vs SHR	0.0063

Figure 6			p value
6a. L-Phe:Tyr ratio	Heart	WKY vs SHR	0.036
		SHR vs acute L-Phe treated	0.017
6b. Adr/Noradr	Heart	WKY vs SHR	0.042
